# Clinical features and CKD-related quality of life in patients with CKD G3a and CKD G3b in China: results from the Chinese Cohort Study of Chronic Kidney Disease (C-STRIDE)

**DOI:** 10.1186/s12882-017-0725-0

**Published:** 2017-10-13

**Authors:** Zhangzhe Peng, Jinwei Wang, Qiongjing Yuan, Xiangcheng Xiao, Hui Xu, Yanyun Xie, Wei Wang, Ling Huang, Yong Zhong, Xiang Ao, Luxia Zhang, Minghui Zhao, Lijian Tao, Qiaoling Zhou, on behalf of the C-STRIDE study group

**Affiliations:** 1 Department of Nephrology, Xiangya Hospital, Central South University, 87 Xiangya Road, Changsha, Hunan 410008 China; 2 Renal Division, Department of Medicine, Peking University First Hospital, Beijing, 100034 China; 3 Institute of Nephrology, Peking University, Beijing, 100034 China; 4 Key Laboratory of Renal Disease, National Health and Family Planning Commission of China; Key Laboratory of Chronic Kidney Disease Prevention and Treatment, Peking University, Ministry of Education, Beijing, 100034 China

**Keywords:** Chronic kidney disease stage 3, Clinical features, Health-related quality of life, Subdivision

## Abstract

**Background:**

This study aimed to compare clinical features and health-related quality of life (HRQoL) in the Chinese chronic kidney disease (CKD) 3 population and determined the necessity of the subdivision of CKD3 in Chinese patients with CKD.

**Methods:**

Participants with stage 3 CKD (18–74 years of age) were recruited at 39 clinical centers located at 28 cities in 22 provinces of China. The sociodemographic status, medical history, anthropometric measurements, and lifestyle behaviors were documented at entry, and blood and urine samples were collected. The estimated glomerular filtration rate was calculated using the CKD-EPI creatinine equation. The HRQoL was evaluated using the kidney disease quality-of-life instrument. A linear regression model was used to estimate the association between HRQoL and CKD stages (G3b vs G3a).

**Results:**

The levels of intact parathyroid hormone, systolic blood pressure, uric acid, and high-density lipoprotein cholesterol were statistically significantly higher, whereas the levels of serum bicarbonate and hemoglobin were statistically significantly lower in the G3b group compared with the G3a group. Compared with CKD G3a group, the proportions of subjects with hyperuricemia and anemia were significantly higher in CKD G3b group (61.4% vs. 52.0% and 26.4% vs. 17.9%, respectively, *P*< 0.01). The HRQoL scores in “physical functioning (PCS)”, “symptoms and problems”, “effects of the kidney disease” and “burden of the kidney disease” were statistically significantly lower in the CKD G3b group compared with the CKD G3a group (90.88 ± 11.05 vs. 89.30 ± 11.52, 88.29 ± 11.94 vs. 86.49 ± 13.45, 55.86 ± 26.40 vs. 52.10 ± 27.64, 46.56 ± 8.16 vs. 44.51 ± 9.22, respectively, *P*< 0.01). Further, CKD G3b was associated with a lower score of physical functioning compared with G3a (regression coefficient =−1.12 [95%CI: −2.23, −0.16]).

**Conclusions:**

The preliminary results of this study suggested that modest differences existed in many important clinical features and KDQoL between patients with G3a and G3b CKD in a Chinese population. Also, a significant association between CKD3 subdivision of the disease and PCS was detected. Although further work is needed, we can speculate based on these results the CKD3 subdivision may be clinically meaningful for Chinese patients with CKD.

**Electronic supplementary material:**

The online version of this article (doi: 10.1186/s12882-017-0725-0) contains supplementary material, which is available to authorized users.

## Background

Chronic kidney disease (CKD) is a dangerous, life-threatening illness defined by declining renal function that gradually progresses to end-stage renal disease (ESRD) [1]. Globally, approximately 200 million patients with CKD have been reported [2]. CKD has also become a leading health problem in China with an estimated 119.5 million patients suffering from the disease as inferred by a recent countrywide survey that demonstrated the 10.8% overall prevalence [3]. This means that more than 50% of patients with CKD in the planet live in China. With a booming economy and aging population, the prevalence is projected to grow continuingly in the near future. CKD is a significant financial burden on global health care systems [4, 5]. The cost of dialysis treatment alone for one patient would be around $14,300 per year, whereas the per capita disposable income is $1210 in urban areas and $375 in rural areas in China [6]. Therefore, the rapidly increasing CKD epidemics cause substantial socioeconomic and public health burden to the Chinese society. Understanding the CKD epidemiology is crucial to control the number of CKD cases in China.

In 2012, the Kidney Disease: Improving Global Outcomes (KDIGO) recommended reclassifying CKD [7], which included a two-dimensional staging of CKD according to the urine albumin-to-creatinine ratio. Furthermore, the classification split CKD stage 3 into two subgroups by applying a cutoff point of eGFR (45 mL/min ⋅ 1.73 m^2^). Hence, subjects with CKD G3a were considered low-risk compared with patients with CKD G3b. This new classification was based on a meta-analysis performed in 45 cohorts involving over 1.5 million participants mainly from developed countries [7]. However, the CKD population in China, the largest developing country, is quite different from that in developed countries. The analysis showed that the stages 3 and 4 CKD prevalence was lower compared with that in developed countries. For example, the prevalence of stage 3 CKD was 1.6% in China compared with 7.7% in the USA and 4.2% in Norway [3]. The rise in obesity and type 2 diabetes mellitus, along with a growing aging population, has exacerbated the financial and medical burden of CKD in China [8, 9]. Due to a lack of resources, however, fewer patients in China receive dialysis compared with those in Western countries. It is still unknown whether this revised classification is suitable for Chinese patients with CKD.

Patients with CKD experience limited health-related quality of life (HRQoL). While the relationship between HRQoL and increased mortality in patients with ESRD is well-established, little is known about the association between early stage of CKD and HRQoL in China. The present study aims to compare clinical features and CKD-related quality of life in the Chinese CKD3 population and determine the necessity of the subdivision of CKD3 in Chinese patients with CKD.

## Methods

### Study population and methods

The design and methods of the Chinese Cohort Study of Chronic Kidney Disease (C-STRIDE) study were published in detail previously [10, 11]. The study was a prospective project which included 39 clinical centers located in 28 cities in 22 provinces of China. All these clinical centers were renal departments from different hospitals. Altogether, 3499 Chinese patients with pre-stage 5 CKD enrolled in the study; 2870 of them completed the required examinations in their entirety. Six hundred twenty nine were excluded from the study due to missing values in key demographic variables or serum creatinine. Of the 2870, a total of 1277 patients with CKD 3 were included in the study analysis.

The baseline data included: detailed demographics; financial, sociological and health care related information; medical and family history including previously taken medications; and questionnaires related to quality of life, health behaviors, and physical activity. Height, weight, waist and hip width, resting blood pressure, and heart rate were collected. Laboratory parameters including complete blood count, serum uric acid, serum creatinine, serum cholesterol, serum calcium and phosphate, fasting blood glucose, high-sensitivity C-reactive protein (Hs-CRP), intact parathyroid hormone (iPTH), urine dipstick, albumin-to-creatinine ratio (ACR), 24-h urine protein, electrolytes, 12-lead surface electrocardiography, echocardiogram and lateral abdominal radiograph were collected for each participant.

The eGFR was determined with the chronic kidney disease epidemiology collaboration equation (CKD-EPI) for Chinese patients with CKD. CKD stage was determined using eGFR and ACR levels. The eGFR stage was graded as follows: G3a, eGFR: 45–59 mL/(min ⋅ 1.73 m^2^) and G3b, eGFR: 30–44 mL/(min ⋅ 1.73 m^2^). The albumin-urine stage was categorized according to an analysis of a spot urine sample: A1 (normoalbuminuria), ACR <30 mg/g creatinine; A2 (microalbuminuria), 30 ≤ ACR < 300 mg/g creatinine; or A3 (macroalbuminuria), ACR ≥300 mg/g creatinine. The A1 stage was sectioned into groups by ACR as follows: A1a, ACR <10 mg/g creatinine and A1b, 10 ≤ ACR < 30 mg/g creatinine.

CKD-related complications that were assessed included hypertension, diabetes, hyperlipidemia, hyperuricemia, anemia, metabolic acidosis, CKD-mineral and bone disorders (CKD-MBD), and cardiovascular disease (CVD). Baseline hypertension was described as a systolic blood pressure > 140 mmHg; a diastolic blood pressure > 90 mmHg (at least three high readings taken within a week of each other); use of antihypertensive medications; or any self-reported history of hypertension. Patients were considered to have diabetes mellitus if they had a fasting glucose ≥7.0 mmol/L; an HbA1c ≥ 6.5%; took insulin or other anti-diabetic medications; or reported a history of diabetes. Hyperlipidemia was defined as a serum concentration with a total cholesterol level ≥ 5.7 mmol/L and an LDL-cholesterol level ≥ 3.6 mmol/L. Hyperuricemia was defined as a serum concentration of uric acid ≥420 μmol/L for men and ≥360 μmol/L for women. Anemia was determined in patients with a hemoglobin level < 120 g/L for men and <110 g/L for women. Metabolic acidosis was identified as a plasma bicarbonate (HCO3^−^) concentration < 22 mmol/L. CKD-MBD was defined as a triad of interrelated abnormalities of serum biochemistry (serum phosphate levels >1.49 mmol/L or <0.87 mmol/L, serum calcium levels >2.37 mmol/L or <2.1 mmol/L, and iPTH >70 pg/mL or <35 pg/mL), bone, and vasculature (plain lateral abdominal x-ray film showed abdominal aortic calcification) associated with CKD. Cardiovascular disease (CVD) was defined as the past occurrence of a myocardial infarction, admittance into a hospital for congestive heart failure, or severe cardiac arrhythmia incidents (resuscitated cardiac arrest, ventricular fibrillation, sustained ventricular tachycardia, paroxysmal ventricular tachycardia, atrial fibrillation or flutter, severe bradycardia, or heart block). Echocardiography showed left ventricular end-systolic diameter (LVESD) >50 mm and/or ejection fraction (EF) <50%.

The Mandarin Chinese version of kidney disease quality of life (KDQoL)-36 instrument, an globally accepted tool, was used for evaluating HRQoL of patients with CKD [12]. The Mandarin Chinese KDQoL-36 was a short, accurate and consistent tool used to determine the quality of life of patients with CKD in China [13]. The disease-related section consisted of 24 items which made up three scales: Symptoms and Problems (12 items), Burden of Kidney Disease (4 items), and Effects of Kidney Disease (8 items). The generic core was the 12-item Short-Form Health Survey (SF-12). The results of the SF-12 instrument were summarized into the Physical Component Summary (PCS) score and the Mental Component Summary (MCS) score. The raw scores were transformed linearly into a range of 0–100 with higher scores representing greater HRQoL [14] (http://www.rand.org/content/dam/rand/pubs/papers/2006/P7994.pdf).

### Statistical methods

Continuous variables were presented as means with standard deviations (SDs) except for serum total cholesterol, duration of CKD, ACR, and Hs-CRP. There were displayed as median (interquartile range) due to high skewness. Categorical variables were presented as counts and proportions. The statistical significance of differences in clinical characteristics was examined using Student *t* test (continuous variables), Wilcoxon signed-rank test (variables with high skewness), and chi-square test (categorical variables). The trend analysis in the proportions among ordinal multiple categorical variables was examined using the Mantel–Haenszel test.

The associations between CKD-related quality of life and CKD stages (G3b vs G3a) were analyzed using a linear regression model. The variables included in the regression model were gender (male vs. female), age (continuous, change by 10 years), high school education (yes vs. no), current smoking (yes vs. no), CVD (yes vs. no), diabetes mellitus (yes vs. no), hyperuricemia (yes vs. no), high blood pressure (yes vs. no), albuminuria (A2 vs. A1 and A3 vs. A1), anemia (yes vs. no), CKD-MBD (yes vs. no), hyperlipidemia (yes vs. no), and metabolic acidosis (yes vs. no). Multiple imputations were performed to handle missing data to reduce the loss of information and account for the uncertainty of missing data in the regression analysis. The regression method was used to impute missing values in a normally distributed continuous variable, predictive mean matching method was used in a skewed distributed continuous variable, and logistic regression was used in a categorical variable with binary or ordinal responses. Twenty-five imputed data sets were generated with the number of iterations of 20. Each imputed dataset was analyzed separately, and then pooled results were obtained. The total percentage of individuals imputed was 32% (407/1277).

All *P* values were two sided. A *P* value <0.05 was deemed to be statistically significant. All analyses were performed using the software program the Statistical Analysis System (SAS) (version 9.4, SAS Institute, CA, USA).

## Results

### Patient characteristics and HRQoL scores

A population of 1277 patients with CKD3 was recruited for the study. The mean age of patients with CKD3 was 51.68 ± 12.81 years, and 60.9% (813) were male. Demographic and laboratory parameters are presented in Table 1, with the patients divided into two groups according to eGFR (mL/(min ⋅ 1.73 m^2^)): CKD G3a, with eGFR range 45–59 (*n* = 499, 39.1%), and CKD G3b, with eGFR range 30–44 (*n* = 778, 60.9%). As shown in Table 1, patients in the CKD G3a group had a mean age of 49.84 ± 12.76 years with 40.8% of men, whereas patients in the CKD G3b group had a mean age of 52.86 ± 12.71 years with 71.1% of men. The high school education level was lower in the CKD G3b group compared with the CKD G3a group (51.8% vs 64.0%, *P* < 0.01). The levels of iPTH, systolic blood pressure, uric acid, and high-density lipoprotein (HDL) cholesterol were statistically significantly higher in the G3b group compared with the G3a group (*P* < 0.05). The levels of serum bicarbonate and hemoglobin were statistically significantly lower in the G3b group compared with the G3a group (*P* < 0.05). The proportions of subjects with hyperuricemia and anemia were statistically significantly higher in the CKD G3b group compared with the CKD G3a group (61.4% vs 52.0%, 26.4% vs 17.9%, respectively, *P* < 0.01). The age- and gender-adjusted prevalence of CKD-related complications in CKD G3b based on the age and gender distribution of CKD G3a was similar to the unadjusted result (Additional file 1: Table S1). The HRQoL scores in “physical functioning,” “symptoms and problems,” “effects of the kidney disease,” and “burden of the kidney disease” were statistically significantly lower in the CKD G3b group compared with the CKD G3a group (*P* < 0.05), but the difference was modest.

**Table 1:** Demographic, laboratory parameters and KDQoL subscales of patients with CKD 3 from C-STRIDE Study

Characteristics	CKD G3a (*n* = 499)	CKD G3b (*n* = 778)	Missing value	*p*-value
Age (years)	49.84 ± 12.76	52.86 ± 12.71	0	<0.001
Men	355 (71.1%)	458 (58.8%)	0	<0.001
Duration of CKD (years)	1 (0,4)	1 (0,4)	0	0.45
Current smoking	199 (40.9%)	313 (41.9%)	43	0.72
Drinkers (≥1 times per day)	15 (3.1%)	23 (3.1%)	55	0.10
High school education (%)	315 (64.0%)	398 (51.8%)	17	<0.001
Body mass index (kg/m2)	24.71 ± 3.37	24.78 ± 4.70	102	0.74
Systolic blood pressure (mmHg)	129.54 ± 16.67	132.01 ± 17.16	143	0.02
Diastolic blood pressure (mmHg)	82.29 ± 15.56	81.65 ± 10.74	143	0.42
Hypertension	400 (80.2%)	631 (81.1%)	0	0.68
Serum total cholesterol (mmol/L)	4.89 (4.00,5.97)	4.68 (3.86,5.79)	75	0.04
Serum LDL cholesterol (mmol/L)	2.67 ± 0.94	2.66 ± 1.00	97	0.82
Serum HDL cholesterol (mmol/L)	1.07 ± 0.31	1.13 ± 0.41	96	0.01
Hyperlipidemia	155 (33.8%)	214 (29.2%)	86	0.10
Serum creatinine (μmol/L)	129.50 ± 18.84	163.27 ± 27.49	0	<0.001
eGFR (mL/min/1.73 m^2^)	51.61 ± 4.24	37.13 ± 4.33	0	<0.001
Fasting Blood glucose (mmol/L)	5.28 ± 1.73	5.41 ± 1.86	77	0.25
Diabetes mellitus	130 (26.1%)	216 (27.8%)	0	0.50
Cardiovascular disease	128 (25.9%)	218 (28.5%)	17	0.32
Uric acid (μmol/L)	402.46 ± 105.51	426.14 ± 111.37	18	<0.001
Hyperuricemia	256 (52.0%)	471 (61.4%)	18	0.001
Albumin	39.81 ± 6.96	39.56 ± 6.60	47	0.52
ACR (mg/g creatinine)	315.37 (63.67,724.88)	340.19 (74.78,860.03)	175	0.19
Hematuria	230 (53.4%)	332 (50.2%)	184	0.30
Hemoglobin	134.17 ± 21.68	127.56 ± 46.83	110	0.004
Anemia	83 (17.9%)	185 (26.4%)	110	<0.001
Serum Bicarbonate	26.49 ± 4.20	25.46 ± 3.83	123	<0.001
Metabolic Acidosis	109 (21.8%)	180 (23.1%)	0	0.59
Serum calcium	2.24 ± 0.22	2.25 ± 0.22	49	0.71
Serum phosphorus	1.17 ± 0.40	1.18 ± 0.25	58	0.84
Serum iPTH	47.95 ± 28.94	59.15 ± 37.74	240	<0.001
CKD-MBD	398 (79.8%)	636 (81.8%)	0	0.38
Hs-CRP	1.5 (0.66,3.33)	1.4 (0.59,3.72)	225	0.51
Symptoms and Problems (S)	90.88 ± 11.05	89.30 ± 11.52	70	0.02
Effects of the Kidney disease (E)	88.29 ± 11.94	86.49 ± 13.45	70	0.02
Burden of the Kidney disease (B)	55.86 ± 26.40	52.10 ± 27.64	70	0.02
SF-12 Physical Functioning (PCS)	46.56 ± 8.16	44.51 ± 9.22	70	<0.001
SF-12 Mental Functioning (MCS)	51.18 ± 8.38	50.2 ± 9.20	70	0.06

Continuous variables are presented as mean ± SD, or median with interquartile ranges. Categorical data are presented as numbers (n) of patients

Abbreviations: *CKD* Chronic kidney disease, *LDL* Low-density lipoprotein, *HDL* High-density lipoprotein, *eGFR* estimated glomerular filtration rate, *ACR* Albumin to creatinine ratio, *CKD-MBD* CKD-mineral and bone disorders, *Hs-CRP* high-sensitivity C-reactive protein

### Proportions of CKD-related complications according to a two-dimensional staging of CKD

Patients with CKD G3a and G3b were classified according to the levels of ACR, and the proportions of CKD-related complications, including hypertension, hyperlipidemia, hyperuricemia, anemia, metabolic acidosis, CKD-MBD, and CVD, were compared. A general trend of higher prevalence of hyperuricemia or anemia through increased ACR levels was observed when the G3b group compared with the G3a group (Table 2, *P* for trend <0.01).

**Table 2:** The percentages of CKD related complications in the groups staged by eGFR and ACR levels

	CKD G3a (*n* = 417)				*P* for trend (G3a)	CKD G3b (*n* = 685)				*P* for trend (G3b)	
	A1a *n* = 39	A1b *n* = 45	A2 *n* = 118	A3 *n* = 215		A1a *n* = 43	A1b *n* = 56	A2 *n* = 226	A3 *n* = 360		*P* for trend (total)
Hypertension	32 (82.1%)	36 (80.0%)	101 (85.6%)	167 (77.7%)	0.37	35 (81.4%)	43 (76.8%)	187 (82.7%)	293 (81.4%)	0.78	0.87
Hyperlipidemia	11 (28.2%)	15 (33.3%)	34 (28.8%)	77 (35.8%)	0.25	6 (14.0%)	11 (19.6%)	51 (22.6%)	127 (35.3%)	<0.001	0.98
Hyperuricemia	21 (53.9%)	17 (37.8%)	61 (51.7%)	117 (54.4%)	0.36	19 (44.2%)	34 (60.7%)	151 (66.8%)	214 (59.4%)	0.55	0.001
Anemia	7 (18.0%)	8 (17.8%)	17 (14.4%)	43 (20.0%)	0.54	12 (27.9%)	9 (16.1%)	40 (17.7%)	104 (28.9%)	0.05	0.001
Metabolic Acidosis	3 (7.7%)	9 (20.0%)	22 (18.6%)	38 (17.7%)	0.34	9 (20.9%)	15 (26.8%)	50 (22.1%)	64 (17.8%)	0.16	0.38
CKD-MBD	30 (76.9%)	33 (73.3%)	95 (80.5%)	168 (78.1%)	0.71	31 (72.1%)	49 (87.5%)	181 (80.1%)	291 (80.8%)	0.63	0.26
CVD	10 (25.6%)	18 (40.0%)	21 (17.8%)	63 (29.3%)	1	11 (25.6%)	18 (32.1%)	71 (31.4%)	98 (27.2%)	0.68	0.56
Quality of Life	40.15 ± 18.69	38.41 ± 17.76	43.87 ± 18.52	42.18 ± 19.16	0.41	37.16 ± 16.27	42.68 ± 17.89	39.54 ± 19.42	38.98 ± 20.71	0.63	0.25

Continuous variables are presented as mean ± SD, or median with interquartile ranges. Categorical data are presented as numbers (n) of patients

Abbreviations: *CKD-MBD* CKD-mineral and bone disorders, *CVD* Cardiovascular disease

### Multiple linear regression

A multiple linear regression analysis was conducted to estimate the relationship between HRQoL and CKD stages (G3b vs G3a). As shown in Table 3, the CKD stage (G3b vs G3a) was independently associated with SF-12 PCS. CKD G3b was associated with a lower score of physical functioning [regression coefficient = −1.12; 95% confidence interval (CI): −2.23 to −0.16] compared with G3a.

**Table 3:** The linear association between chronic kidney disease G3a/G3b and KDQoL subscales

Risk factor	Regression coefficient (95% confidence interval)^a^	*p*-value
Symptoms and Problems (S)	−0.91 (−2.26,0.43)	0.18
Effects of the Kidney Disease (E)	−1.38 (−2.94,0.17)	0.08
Burden of the Kidney Disease (B)	−3.00 (−6.25,0.26)	0.07
SF-12 Physical Functioning (PCS)	−1.12 (−2.23,-0.16)	0.02
SF-12 Mental Functioning (MCS)	−1.00 (−2.08,0.06)	0.07

^a^Adjusted for age, gender, education level, current smoking, hypertension, diabetes, cardiovascular disease, diabetes mellitus, hyperuricemia, high blood pressure, albuminuria, anemia, CKD-mineral and bone disorders, hyperlipidemia and metabolic acidosis

## Discussion

The present study investigated clinical features and CKD-related quality of life in patients with CKD G3a and CKD G3b from the C-STRIDE Study in China. The findings could be summarized as follows: Significant differences were observed in many important clinical features, including the levels of iPTH, systolic blood pressure, uric acid, HDL cholesterol, serum bicarbonate, and hemoglobin, and the prevalence of hyperuricemia and anemia between the CKD G3b and CKD G3b groups. The HRQoL scores were statistically significantly higher in patients with CKD G3b group compared with scores of patients with CKD G3a. CKD G3b was associated with a lower score of SF12 physical functioning.

Stage 3 CKD is the first stage that can be identified from the serum creatinine test alone and accounts for the vast majority of people now being detected and labeled with CKD on general practice disease registers [15]. In a national study of 47,204 Chinese adults, the adjusted prevalence of stage 3 CKD was 1.6% (95% CI: 1.4–1.8) compared with 0.1% (95% CI 0.06–0.2) in stage 4 and 0.03% (95% CI 0.01–0.05) in stage 5 [3]. Consistently, patients with stage 3 CKD outnumber those with stage 4 almost 20-fold [16]. The clinical implications of patients with stage 3 are less clear compared with the patients with higher CKD stages, whose clinical features and outcomes of CKD are well understood. Many scientists recommend subdividing stage 3 into stages G3a (45–59 mL/min) and G3b (30–44 mL/min), since these subgroups may associate with unique clinical patterns [17, 18]. In a meta-analysis conducted by KDIGO in 2009, a steep rise in risk of mortality and kidney outcomes with lower eGFR in the range of 30–59 mL/(min ⋅ 1.73 m^2^) was observed, which was consistent with the proposal to divide stage 3 into two stages [7].

The results of this study confirmed different clinical features and HRQoL between patients with CKD G3a and G3b in China. Consistently with our findings, Ito et al., in a cross-sectional study of 2088 Japanese patients with type 2 diabetes mellitus, observed that the percentage of patients with diabetic retinopathy, neuropathy and other complications was statistically higher in patients with CKD G3b than in patients with CKD G3a [19]. A group of 390 Chinese patients with immunoglobulin A nephropathy (IgAN) showed that grouping IgAN patients with CKD stage 3 by G3a and G3b was useful in evaluating risk of disease progression and assessing prognosis of patients [20].

Previous results have shown that albuminuria correlates with the risk of adverse outcomes independently of eGFR [21]. Thus, the revised classification proposed by KDIGO in 2012 recommended a two-dimensional staging of CKD according to the urine ACR. In the present study, a general trend of higher prevalence of hyperuricemia or anemia through increased ACR levels was detected when the G3b patients were compared with the G3a patients. However, the prevalence of CKD-related complications was not found to be much higher in the macro-albuminuria group than that in the non-albuminuria group. Consistently, higher ACR was not significantly associated with a higher prevalence of CKD-related complications within the CKD G3a or CKD G3b group. The explanation for this observation might be that macro-albuminuria with fewer signs or symptoms did not have an influence on CKD-related complications in patients in the early stage of CKD. A cohort study is ongoing to reveal the association among ACR and CKD-related complications in Chinese patients with CKD.

Functioning in everyday activities and well-being are essential when considering chronic disease heath care. Previous studies found that patients with CKD undergo a barrage of symptoms and significant impairments in the HRQoL [22]. All HRQoL dimensions declined significantly with CKD stage, with the lowest scores in CKD 5 compared with healthy controls. Besides, the impaired HRQoL has been demonstrated to predict mortality and cardiovascular events [23, 24]. While HRQoL and increased mortality in patients with ESRD has been well-studied, less is understood about the link between the early stage of CKD and HRQoL in China.

These findings suggested that Chinese patients with CKD3 experience an overall burden of symptoms, impaired physical well-being, and low HRQoL. Patients with CKD G3b had lower scores on all HRQoL dimensions compared with patients with CKD G3a although the differences were small (1 or 2 points) between the two groups. Similar to the findings of the present study, other studies have shown that the differences in scores were quite small but still significant in patients with the early stage of CKD, indicating that KDQoL subscales were sensitive to differences in the CKD stage [14, 25]. More importantly, the change in KDQoL score predicted continued survival, hospitalization, and dialysis-attendance compliance in hemodialysis patients. Each 5-point increase in PCS scores was related to a 10% increase in survival and 6% fewer length of stay in the hospital. Each 5-point improvement in the MCS score was linked to 2% fewer hospital days [26]. After controlling for several variables, the CKD stage (G3b vs G3a) was only significantly associated with PCS. The results of the present study confirmed findings from other studies which found that eGFR values around 45 mL/(min ⋅ 1.73 m^2^) are the threshold for the decrease in the HRQoL, especially in PCS [27].

The present study included the following limitations. Only relationship between variables, and not causal relationships, could be surmised due to the cross-sectional study design. Thus, longitudinal studies that consider qualitative evaluations ought to be conducted to seek a deeper understanding of the clinical difference in the prognosis of patients with CKD3 in China. When interpreting the results, it should be considered that the SF-12 used in the present study did not cover all measures in the HRQoL for patients with CKD, such as sleep, sexual and cognitive functioning.

## Conclusions

In the present study, modest differences existed in many important clinical features and KDQoL between patients with G3a and G3b CKD in a Chinese population. Also, a significant association between CKD3 subdivision of the disease and PCS was detected. Although further work is needed, we can speculate based on these results the CKD3 subdivision may be clinically meaningful for Chinese patients with CKD.

## Additional file

Additional file 1: Table S1. The Age and sex adjusted prevalence of CKD related Complications in CKD G3b based on the age and gender distribution of CKD G3a. (12 kb)

## Abbreviations

ACR: Albumin-to-creatinine ratio
CKD: Chronic kidney disease
CKD-EPI: Chronic kidney disease epidemiology collaboration equation
CKD-MBD: CKD-mineral and bone disorders
C-STRIDE: Chinese cohort study of chronic kidney disease
CVD: Cardiovascular disease
DM: Diabetes mellitus
eGFR: Estimated glomerular filtration rate
ESRD: end-stage renal disease
HBP: High blood pressure
HDL: High-density lipoprotein
HRQoL: health-related quality of life
Hs-CRP: High-sensitivity C-reactive protein
iPTH: Intact parathyroid hormone
IQR: Interquartile range
KDOQI: Kidney disease outcomes quality initiative
KDQoL: Kidney disease quality of life
LDL: Low-density lipoprotein
LVESD: Left ventricular end-systolic diameter
MCS: Mental summary scores
PCS: Physical summary scores
SDs: Standard deviations; UA, uric acid
