# Supplementary material for: Clinical features and CKD-related quality of life in patients with CKD G3a and CKD G3b in China: results from the Chinese Cohort Study of Chronic Kidney Disease (C-STRIDE)
Source: BMC Nephrol. 2017 Oct 13;18:311. doi: 10.1186/s12882-017-0725-0 (PMC5640906; doi:10.1186/s12882-017-0725-0)
Supplement: Additional file 1: Table S1. — The Age and sex adjusted prevalence of CKD related Complications in CKD G3b based on the age and gender distribution of CKD G3a. (12 kb) [file 12882_2017_725_MOESM1_ESM.docx]

Additional file 1 Table S1 The Age and sex adjusted prevalence of CKD related Complications in CKD G3b based on the age and gender distribution of CKD G3a

| Characteristics | CKD G3a(n=499) | CKD G3b(n=778) |
| --- | --- | --- |
| Hypertension | 80.2% | 81.8% |
| Hyperlipidemia | 33.8% | 29.1% |
| Hyperuricemia | 52.0% | 62.0% |
| Anemia | 17.9% | 25.8% |
| Metabolic Acidosis | 21.8% | 24.2% |
| CKD-MBD | 79.8% | 81.4% |
| Cardiovascular disease | 25.9% | 29.2% |

Abbreviations: CKD-MBD: CKD-mineral and bone disorders
